# Knockdown of PLC-gamma-2 and calmodulin 1 genes sensitizes human cervical adenocarcinoma cells to doxorubicin and paclitaxel

**DOI:** 10.1186/1475-2867-12-30

**Published:** 2012-06-18

**Authors:** Anthony Stanislaus, Athirah Bakhtiar, Diyana Salleh, Snigdha Tiash, Tahereh Fatemian, Sharif Hossain, Toshihiro Akaike, Ezharul Hoque Chowdhury

**Affiliations:** 1 Faculty of Medicine and Health Science, International Medical University (IMU), No. 126, Jalan 19/155B, Bukit Jalil, Kuala Lumpur, 57000, Malaysia; 2 Jeffrey Cheah School of Medicine and Health Sciences, Monash University Sunway Campus, Jalan Lagoon Selatan, Bandar Sunway, Selangor Darul Ehsan, Malaysia; 3 Department of Biomolecular Engineering, Graduate School of Bioscience and Biotechnology, Tokyo Institute of Technology, 4259 Nagatsuta-cho, Midori-ku, Yokohama, 226-8501, Japan

**Keywords:** Carbonate apatite, Nanoparticle, siRNA, PLC-gamma-2, Calmodulin 1, Cervical cancer, Cisplatin, Doxorubicin, Paclitaxel

## Abstract

**Background:**

RNA interference (RNAi) is a powerful approach in functional genomics to selectively silence messenger mRNA (mRNA) expression and can be employed to rapidly develop potential novel drugs against a complex disease like cancer. However, naked siRNA being anionic is unable to cross the anionic cell membrane through passive diffusion and therefore, delivery of siRNA remains a major hurdle to overcome before the potential of siRNA technology can fully be exploited in cancer. pH-sensitive carbonate apatite has recently been developed as an efficient tool to deliver siRNA into the mammalian cells by virtue of its high affinity interaction with the siRNA and the desirable size distribution of the resulting siRNA-apatite complex for effective cellular endocytosis. Moreover, internalized siRNA was found to escape from the endosomes in a time-dependent manner and efficiently silence gene expression.

**Results:**

Here we show that carbonate apatite-mediated delivery of siRNA against PLC-gamma-2 (PLCG2) and calmodulin 1 (CALM1) genes has led to the sensitization of a human cervical cancer cell line to doxorubicin- and paclitaxel depending on the dosage of the individual drug whereas no such enhancement in cell death was observed with cisplatin irrespective of the dosage following intracellular delivery of the siRNAs.

**Conclusion:**

Thus, PLCG2 and CALM1 genes are two potential targets for gene knockdown in doxorubicin and paclitaxel-based chemotherapy of cervical cancer.

## Background

Genes are transcribed into mRNAs and subsequently translated into proteins to carry out the major functions within a cell and the mutations in certain genes leading to their suppression or overexpression are usually responsible for both acquired and genetic diseases. Delivery of functional gene(s) or gene-silencing element(s) could be the potential options in restoring the normal functions of the cell. RNA interference (RNAi) that can selectively silence mRNA expression in cell cytoplasm can be utilized to develop new drugs against target therapeutic genes [1-5]. RNAi can be harnessed for selective gene inhibition in two different routes: 1) cytoplasmic delivery of short interfering RNA (siRNA) for directly breaking down the specific mRNA and 2) nuclear delivery of gene expression cassettes to express a short hairpin RNA (shRNA) which is further processed by cellular machinery to siRNA in the cytoplasm [6]. However, siRNA, a synthetic RNA duplex of 21–23 nucleotides, is more advantageous than shRNA because of the difficulty in the construction of a shRNA expression system [6], and the requirement of the expression system to overcome the nuclear barrier for shRNA expression [7]. siRNA in the cytoplasm of the cells incorporates into a multiprotein RNA-induced silencing complex (RISC) and is unwound into single-stranded RNAs by Argonaute 2, a multifunctional protein within the RISC, forming antisense strand-associated RISC in order to guide and selectively degrade the complementary mRNA with the help of Argonaute-2 [8]. Perfect hybridization between the antisense strand of siRNA and the target mRNA leads to degradation of the mRNA near the center of the target-siRNA duplex [8]. However, the strong anionic phosphate backbone with consequential electrostatic repulsion from the anionic cell membrane is an obstacle to the passive diffusion of siRNA across the membrane [9]. The hydrophobic lipid bilayer could pose an additional barrier to the hydrophilic siRNA. Moreover, naked siRNA can be degraded by the plasma nucleases and even subjected to renal elimination due to its small size before reaching the target site *in vivo*[10,11]. A number of existing non-viral vectors have been developed for intracellular siRNA delivery with limited efficacy [8]. Usually, a non-viral vector being cationic can electrostatically bind with an anionic siRNA to form a stable complex, thus protecting it from nuclease-mediated degradation, enabling it to cross the plasma membrane through endocytosis and finally facilitating its endosomal escape [8].

Cancer is a complex disease responsible for millions of deaths worldwide and despite remarkable efforts made in the last decades limited successes have been achieved so far to cure various types of cancer. Clinical efficacy of current chemotherapeutic drugs are often limited owing to to their toxic effects on normal cells and the patients can tolerate only the doses which are therapeutically insufficient, thus leading to chemoresistance and subsequent tumor recurrence [12]. Since cancer is the result of overexpression or suppression of signaling pathways aiding cancer cell survival and proliferation, non-viral vector-mediated delivery of siRNAs specific for the genes of pathways, to cancer cells would be the potential treatment options that might additionally render cancer cells extremely sensitive to cytotoxic chemotherapy [11]. Among the signalling cascades, MAP kinase, PI-3 knase and Ca^2+^-calmodulin pathways are extensively involved in proliferation and survival of various cancer cells [13-15]. On the other hand, conventionally used chemotherapy drugs induce apoptosis of cancer cells by interfering with the major cellular functions which might have some of cross-talk with the components of cell proliferation/survival pathways. siRNA-mediated knock-down of the genes encoding the enzymes of those pathways, therefore, might not only slow down the growth of cancer cells, but also sensitize them to anti-cancer drugs.

In Ca^2+^-calmodulin pathway, stimulation with growth factor either G protein-coupled receptors or receptor tyrosine kinases activates the phospholipase C (PLC) enzyme, which, in turn, hydrolyses the membrane phospholipid, phosphatidylinositol 4, 5 bisphosphate (PIP2) to diacylglycerol (DAG) and inositol (1,4,5) trisphosphate (IP3). DAG activates PKC while IP3 binds to its receptor on the endoplasmic reticulum allowing diffusion of Ca^2+^ from the ER to increase intracellular [Ca^2+^[16]. The released Ca^2+^ binds to calmodulin (CaM) and Ca^2+^/CaM functions as an allosteric activator of a considerable number of protein kinases regulating cell proliferation and apoptosis [17].

Recently, we have developed an efficient siRNA delivery system based on some unique properties of carbonate apatite- electrostatic affinity for binding anionic siRNA, ability of preventing crystal growth for generation of nano-size particles for efficient endocytosis and fast dissolution kinetics in endosomal acidic compartments to facilitate the release of siRNA from the particles as well as from the endosomes, leading to the efficient silencing of reporter gene expression. Moreover, nanoparticle-assisted delivery of validated siRNA against cyclin B1 resulted in the significant inhibition of cancer cell growth [18,19].

Here we show that carbonate apatite-mediated delivery of siRNA against PLC-gamma-2 (PLCG2) and calmodulin 1 (CALM1) genes sensitized a human cervical cancer cell line (HeLa cell) to doxorubicin- and paclitaxel-induced cell death depending on the doses of the drugs while no such synergistic effect was observed with cisplatin, another commonly used chemotherapy drugs.

## Results and discussion

### Roles of PLCG2 and CALM1 in the proliferation/survival of cervical cancer cells

**Figure 1:**
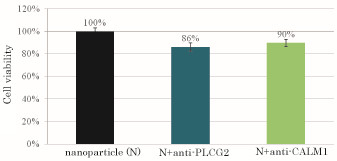
**Effects of silencing of PLCG2 and CALM1 expression on cancer cell viability**. 50,000 of HeLa cells from the exponentially growth phase were seeded in each of the wells of a 24-well plates the day before the siRNA/apatite complexes were prepared by mixing 3 μl of 1 M CaCl_2_ with 10 nM of siRNA in 1 ml of fresh serum-free HCO_3_^-^ (44 mM)-buffered DMEM medium (pH 7.5) and incubating at 37 °C for 30 min. The medium containing the siRNA/apatite complexes supplemented with 10% FBS had been added onto the rinsed cells before the cells were cultured consecutively for 48 h and the assessment on cell viability was carried out. Each experiment was done in triplicate and the data represent mean value ± SE (n = 3).

In order to investigate the potential roles of PLCG2 and CALM1 in the proliferation or survival of HeLa cells that express both of the proteins [20-23], specific validated siRNA (10 mM) against PLCG2 or CALM1 mRNA was added together with Ca^2+^ (3 mM) to the bi-carbonate-buffered DMEM prior to the incubation at 37 °C for 30 min to form carbonate apatite/siRNA complexes. Figure 1 shows the cell viability as assessed by MTT assay following consecutive 48 h incubation of HeLa cells with the apatite complexes carrying either anti-PLCG2 or anti-CALM1 siRNA. Almost 10% of the cells were killed due to the silencing of either PLCG2 or CALM1 gene expression indicating that PLCG2, an upstream molecule and CALM1, a downstream molecule of Ca^2+^-calmodulin pathway are critically involved in the proliferation or survival of HeLa cells.

While both of the siRNAs were validated by the manufacturer (QIAGEN) using quantitative RT-PCR to confirm their knockdown efficiency of 82%, the relatively low efficacy of either treatment in killing cancer cells as compared to the free particles (positive control) was possibly due to the constitutive expression of the genes in spite of the cleavage of substantial amount of the respective mRNAs and the active roles being played by MAP kinase and PI-3 kinase pathways in cell survival or proliferation.

### Influences of PLCG2 and CALM1 gene knockdown on cisplatin-induced cell toxicity

**Figure 2:**
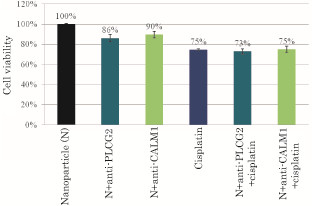
**Effects of silencing of PLCG2 and CALM1 expression on viability of cancer cells under higher dose of cisplatin**. 50,000 of HeLa cells from the exponentially growth phase were seeded in each of the wells of a 24-well plates the day before the siRNA/apatite complexes were prepared by mixing 3 μl of 1 M CaCl_2_ with 10 nM of siRNA in 1 ml of fresh serum-free HCO_3_^-^ (44 mM)-buffered DMEM medium (pH 7.5) and incubating at 37 °C for 30 min. The medium containing the siRNA/apatite complexes supplemented with 10% FBS had been added onto the rinsed cells either with or without 1 μM of cisplatin before the cells were cultured consecutively for 48 h and the assessment on cell viability was carried out. Each experiment was done in triplicate and the data represent mean value ± SE (n = 3).

Cisplatin is one of the most effective anti-cancer drugs for solid tumors, including ovarian, testicular, cervical, and small cell lung cancers [24,25]. Treatment of HeLa cells with 1 μM of cisplatin for 2 consecutive days caused 25% of cell death compared with particles only (Figure 2) and almost same level of cell death was observed for the treatment where both apatite/siRNA complexes and cisplatin were incubated together with the cells for the same period of time, suggesting an additive effect on cell death probably owing to the lack of cross-talk(s) between the pathways of Ca^2+^-calmodulin signaling and cisplatin-mediated toxicity.

**Figure 3:**
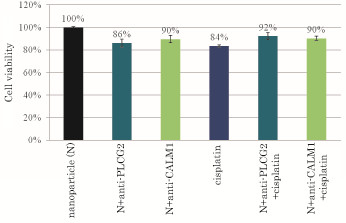
**Effects of silencing of PLCG2 and CALM1 expression on viability of cancer cells under lower dose of cisplatin**. 50,000 of HeLa cells from the exponentially growth phase were seeded in each of the wells of a 24-well plates the day before the siRNA/apatite complexes were prepared by mixing 3 μl of 1 M CaCl_2_ with 10 nM of siRNA in 1 ml of fresh serum-free HCO_3_^-^ (44 mM)-buffered DMEM medium (pH 7.5) and incubating at 37 °C for 30 min. The medium containing the siRNA/apatite complexes supplemented with 10% FBS had been added onto the rinsed cells either with or without 200 nM of cisplatin before the cells were cultured consecutively for 48 h and the assessment on cell viability was carried out. Each experiment was done in triplicate and the data represent mean value ± SE (n = 3).

On the contrary, the combined treatment with apatite/anti-PLCG2 siRNA complex and a lower dose of cisplatin (200 nM) led to the enhancement of cell viability compared with apatite/anti-PLCG2 siRNA or cisplatin (Figure 3), indicating that cisplatin at that particular dose might activate another form of PLC [26] or activate MAP kinase/PI-3 kinase signaling cascades, leading to the enhanced cell growth in the absence of PLCG2 . Silencing of PLCG2 gene promoted more cell growth than silencing of CALM1 gene at that lower dose of cisplatin probably because PLCG2 is more upstream to and therefore, more important regulator than CALM1 in Ca^2+^-calmodulin signaling.

### Influences of PLCG2 and CALM1 gene knockdown on doxorubicin-induced cell toxicity

**Figure 4:**
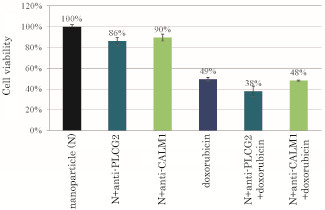
**Effects of silencing of PLCG2 and CALM1 expression on viability of cancer cells under higher dose of doxorubicin**. 50,000 of HeLa cells from the exponentially growth phase were seeded in each of the wells of a 24-well plates the day before the siRNA/apatite complexes were prepared by mixing 3 μl of 1 M CaCl_2_ with 10 nM of siRNA in 1 ml of fresh serum-free HCO_3_^-^ (44 mM)-buffered DMEM medium (pH 7.5) and incubating at 37 °C for 30 min. The medium containing the siRNA/apatite complexes supplemented with 10% FBS had been added onto the rinsed cells either with or without 1 μM of doxorubicin before the cells were cultured consecutively for 48 h and the assessment on cell viability was carried out. Each experiment was done in triplicate and the data represent mean value ± SE (n = 3).

Doxorubicin is another chemotherapy drug widely used for the treatment of a variety of cancers including cervical cancer [27,28]. Doxorubicin which killed almost 50% of the cells at 1 μM concentration of the drug (Figure 4) seems to be more potent than cisplatin which killed 25% of the cells at the same dose (Figure 2) following continuous 2 day incubation with HeLa cells. Silencing of PLCG2 gene following intracellular delivery of apatite/anti-PLCG2 siRNA, clearly sensitized the cells to doxorubicin at that particular concentration (1 μM) killing more than 60% of the cells due to the synergistic effect of the drug and the gene knockdown. This could be due to the activation of Ca^2+^-calmodulin pathway [29] by doxirubicin- an effect that might have hindered the cytotoxic effect of doxorubicin and therefore, targeted cleavage of PLC mRNA or to some extent calmodulin 1 mRNA resulted in blocking of the Ca-calmodulin pathway and inhibition of cell growth or proliferation (Figure 4), thus synergistically enhancing the cancer cell apoptosis in presence of doxorubicin.

**Figure 5:**
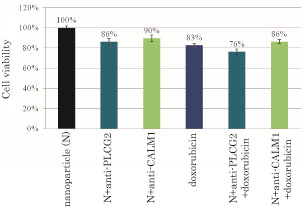
**Effects of silencing of PLCG2 and CALM1 expression on viability of cancer cells under lower dose of doxorubicin**. 50,000 of HeLa cells from the exponentially growth phase were seeded in each of the wells of a 24-well plates the day before the siRNA/apatite complexes were prepared by mixing 3 μl of 1 M CaCl_2_ with 10 nM of siRNA in 1 ml of fresh serum-free HCO_3_^-^ (44 mM)-buffered DMEM medium (pH 7.5) and incubating at 37 °C for 30 min. The medium containing the siRNA/apatite complexes supplemented with 10% FBS had been added onto the rinsed cells either with or without 200 nM of doxorubicin before the cells were cultured consecutively for 48 h and the assessment on cell viability was carried out. Each experiment was done in triplicate and the data represent mean value ± SE (n = 3).

Similar finding was observed after intracellular delivery of anti-PLCG2 siRNA and 200 nM of doxorubicin, whereas delivery of anti-CALM1 siRNA did not result in a synergistic effect in combination with doxorubicin (200 nM) (Figure 5) probably because of CALM1 location more downstream to PLCG2 in the pathway.

### Influences of PLCG2 and CALM1 gene knockdown on paclitaxel-induced cell toxicity

**Figure 6:**
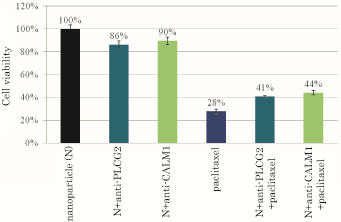
**Effects of silencing of PLCG2 and CALM1 expression on viability of cancer cells under higher dose of paclitaxel**. 50,000 of HeLa cells from the exponentially growth phase were seeded in each of the wells of a 24-well plates the day before the siRNA/apatite complexes were prepared by mixing 3 μl of 1 M CaCl_2_ with 10 nM of siRNA in 1 ml of fresh serum-free HCO_3_^-^ (44 mM)-buffered DMEM medium (pH 7.5) and incubating at 37 °C for 30 min. The medium containing the siRNA/apatite complexes supplemented with 10% FBS had been added onto the rinsed cells either with or without 1 μM of paclitaxel before the cells were cultured consecutively for 48 h and the assessment on cell viability was carried out. Each experiment was done in triplicate and the data represent mean value ± SE (n = 3).

Paclitaxel as a microtubule stabilizer is used for the treatment for various cancers including cervical cancer in combination with cisplatin and other cancer drug(s) [30,31]. As shown in Figures 6,1 μM paclitaxel when incubated with HeLa cells continuously for 2 days, caused more than 70% of the cells to death, indicating that paclitaxel is the most effective of the 3 drugs used in the study. However, the combined treatment of the apatite/siRNA complexes possessing either anti-PLCG2 or anti-CALM1 siRNA and paclitaxel (1 μM) resulted in reduction of the total cell death by almost 10%. The could be explained by the notion that silencing of the PLCG2 and CALM1 genes ends up with the down-regulation of Ca^2+^/calmodulin signaling and the decline in the level of Ca^2+^/calmodulin-dependent protein kinases (CaMKs). Since CAMKs regulate microtubule dynamics by phosphorylation of the microtubule regulator stathmin [32], the overall effect of gene knockdown might cause disruption of microtubule dynamics, thus preventing paclitaxel to stabilize all of the microtubules and more effectively arrest the cell cycle for induction of apoptosis at that relatively higher concentration of the drug.

**Figure 7:**
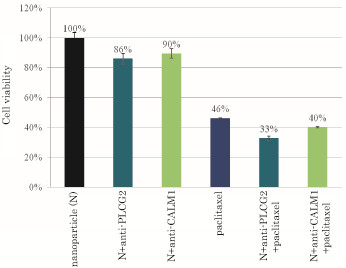
**Effects of silencing of PLCG2 and CALM1 expression on viability of cancer cells under lower dose of paclitaxel**. 50,000 of HeLa cells from the exponentially growth phase were seeded in each of the wells of a 24-well plates the day before the siRNA/apatite complexes were prepared by mixing 3 μl of 1 M CaCl_2_ with 10 nM of siRNA in 1 ml of fresh serum-free HCO_3_^-^ (44 mM)-buffered DMEM medium (pH 7.5) and incubating at 37 °C for 30 min. The medium containing the siRNA/apatite complexes supplemented with 10% FBS had been added onto the rinsed cells either with or without 200 nM of doxorubicin before the cells were cultured consecutively for 48 h and the assessment on cell viability was carried out. Each experiment was done in triplicate and the data represent mean value ± SE (n = 3).

On the contrary, when the concentration of paclitaxel was lowered to 200 nM, silencing of PLCG2 or CALM1 gene expression was associated with a robust decrease in cell viability demonstrating a synergistic effect of the drug action and the gene knockdown on cell proliferation or survival (Figure 7). Since Ca^2+^/CaM promotes cell proliferation by facilitating G_2_/M transition, M phase progression, and exit from mitosis [15] while microtubules induces apoptosis by arresting G2/M phase [33], silencing of either PLCG2 or CALM1 gene in presence of paclitaxel resulted in complete arrest of the cell cycle.

## Conclusions

PLCG2 and CALM1 of Ca^2+^-calmodulin signalling pathways are the two potential targets for gene knockdown in doxorubicin and paclitaxel-based chemotherapy of cervical cancer. Therefore, pre-clinical study in animal models of cervical cancer should be carried out through tumor-targeted delivery of anti-PLCG2 or CALM1 siRNA in combination with passively diffusible anti-cancer drugs.

## Methods

### Reagents

MTT (3-(4,5-Dimethylthiazol-2-yl) -2,5-diphenyl tetrazolium bromide) and DMEM were purchased from Molecular Probes, Sigma and Gibco BRL, respectively. Validated siRNAs against PLCG2 (Target sequenc 5'-GACGACGGTTGTGAATGATAA-3') and CALM1(Target sequence 5'-CGGCAACTTACACACATTGAA-3') were obtained from Qiagen. Upon delivery in the lyophilized form, the siRNAs were diluted to obtain a 20 μM solution using RNAse-free water and allocated into multiple reaction tubes for storage at -20^o^ C as repeated thawing might affect siRNA’s silencing efficiency.

### Cell culture

HeLa cells were cultured in 25-cm^2^ flasks in Dulbecco’s modified Eagle’s medium (DMEM, Gibco BRL) supplemented with 10% fetal bovine serum (FBS), 50 μg penicillin ml-1, 50 μg streptomycin ml-1 and 100 μg neomycin ml-1 at 37 °C in a humidified 5% CO_2_-containing atmosphere.

### Formation of siRNA/carbonate apatite complexes and transfection of cells

Cells from the exponentially growth phase were seeded at 50,000 cells per well into 24-well plates the day before transfection. 3 μl of 1 M CaCl_2_ was mixed with 10 nM of siRNA in 1 ml of fresh serum-free HCO_3_^-^ (44 mM)-buffered DMEM medium (pH 7.5), followed by incubation at 37 °C for 30 min for complete generation of siRNA/carbonate apatite particles [18,19]. 10% FBS and (depending on the experimental conditions) 0.2 to 1 μM drugs (cisplatin, doxorubicin, paclitaxel) had been mixed with the medium containing the siRNA/apatite complexes before the medium was added onto the rinsed cells. The cells were subsequently cultured for 48 h prior to the assessment on cell viability [18,19].

### Cell viability assessment with MTT assay

30 μl of MTT solution (5 mg/ml) was added onto the cells in each well of the 24-well plate and incubated for 4 hr at 37 °C. 0.5 ml of DMSO was added after removal of the medium from each well to resolve the crystals, followed by incubation for 5 min at 37 °C. Absorbance was measured in a micro plate reader at 570 nm with a reference wavelength of 630 nm. Each experiment was done in triplicate with the data representing mean value ± SE (n = 3) and being statistically significant (< 0.05).

## Abbreviations

PLCG2, PLC-gamma-2; CALM1, Calmodulin 1; PIP2, Phosphatidylinositol 4, 5 bisphosphate; DAG, Diacylglycerol; IP3, Inositol (1,4,5) trisphosphate; CaM, Calmodulin; PLC, Phospholipase C; DMEM, Dulbecco's Modified Eagle’s Medium; RNAi, RNA interference; siRNA, Small interfering RNA; shRNA, Short hairpin RNA; FBS, Fetal bovine serum; MTT, (3-(4,5-Dimethylthiazol-2-yl) -2,5-diphenyl tetrazolium bromide).

## Competing interests

The authors declare that they have no competing interests.

## Authors’ contributions

AS has originally planned the project in close discussion with EHC and TA and finally carried out the experiments in collaborations with MJC, APK and SH. All authors read and approved the final manuscript.
